# German Psychological Literature
*Allgemeine Zeitschrift für Psychiatrie unter der Redaction von Damerow, Flemming und Roller. Berlin. 1851. Achter Band, Erstes und Zweetes Heft.


**Published:** 1852-01-01

**Authors:** 


### Art. III.—GERMAN PSYCHOLOGICAL LITERATURE.*

We have before us parts 1 and 2, vol. viii., of the 'Journal of Psychiatry,' an analysis of which we purpose laying before our readers. We are anxious, in the first instance, to direct attention to two papers, one by *Dr. G. H. Bergmann*, entitled 'Pathological Remarks, characterising the different organs of the brain and their functions;' and the other, by *Dr. W. Nasse*, upon the 'relation of the faculty of speech to the anterior lobes of the brain.'

The cases related by Dr. Bergmann illustrate the possibility of carrying minuteness of detail into excess, and of obscuring the truth by immersing it in irrelevant matter. The error, however, flows from a good source; namely, a desire of preserving an accurate record of every symptom noticed during life, and of every appearance observed in the examination after death. A case so completed may be referred to by all persons wishing to form upon it their own conclusions; but at the same time, when observations of importance are thrown together with remarks of no value considerable discrimination is required to separate the ore from the dross.

We give, as an example, the narration of the following case, and the record of the morbid appearances discovered upon dissection.

R., æt. 48, a corpulent man, of full habit, with permanently red complexion, dark hair and eyebrows, and with a sullen domineering look, died 1828. Two days before his death Dr. Bergmann was called to see him by the usual medical attendant, and he found him, at five in the afternoon, sitting upon a sofa in a state of stupefaction, vacant and silent, although he could still utter certain words. The face was blueish-red, and bloated; his large eyes fixed, staring, and devoid of soul or expression. The pulse was not quick, but full and hard; the same beat was felt in the carotids. As Dr. Bergmann was pressing the left carotid to investigate the nature of the beat, the patient fell down suddenly, completely insensible; the pulse ceased for a couple of minutes, the head fell back, the limbs contracted convulsively, foam issued from the mouth, and the eyes rolled upwards. Dr. Bergmann concludes there was no doubt but that the pressure upon the carotid artery caused this accident, by temporarily arresting so important a part of the cerebral circulation. We will not here enter upon the question as to how far pressure upon the internal jugular vein would excite these symptoms, by checking the return of a current of venous blood from a brain affected

* Allgemeine Zeitschrift für Psychiatrie unter der Redaction von Damerow, Flemming und Roller. Berlin. 1851. Achter Band, Erstes und Zweetes Heft.

with apoplexy, and it is indisputable that no pressure can be made upon the one vessel in the neck without its also acting upon the other, which lies in immediate contact. The abstraction of blood afforded to the patient some relief; previously he had not been in a condition to give intelligible replies to questions, the names of persons and of things being blotted out of his memory: he called every object by the word 'thing,' and could not express himself better. Upon the following night he had two convulsive attacks, between epilepsy and apoplexy, and, sinking into a state of yet more complete insensibility, he died the following day.

The body, examined 24 hours after death, emitted an offensive odour. "The abdomen was blown up, and marked with the greenish spots of decomposition. Under the integument there was a thick layer of fat, and there were large adipose accumulations in the greater and lesser omenta, in the mesentery of the whole alimentary canal, including the vermiform process."

We are next told that the "pancreas was narrow and a little hard; the liver moderately large, but nothing in excess, somewhat paler than natural both on its external and internal surface; the gall-bladder was filled with greenish bile. The spleen was, so to say, diseased, long, narrow, soft and yielding, like a damask-plum, surrounded by fat, and united to surrounding textures."

What information are we to derive from this somewhat prosy account, except that the body of a patient, described during life as corpulent, exhibited upon examination after death considerable accumulations of fat in the usual situations?

"In the chest there was much water; the lungs were of dark blue colour but natural; there was a considerable collection of fat about the heart, which was large; the ventricles were dilated, especially the right; the muscular substance and the valves exhibited a certain amount of relaxation and softness."

The presence of fluid in the chest, and the morbid condition of the heart, are points of high interest, and yet they are dismissed without a word of allusion to the quantity of fluid in the first instance, or the nature of the degeneration of the muscular substance in the second. Much fewer words are here used than in the description of the amount of abdominal fat. But it is with the account of the morbid condition of the brain that we find greatest reason to complain.

"The skull-cap, though flat, was natural. Under the pia-mater, over both hemispheres, there was effused a thick gelatinous fluid, which in the base of the brain was of more watery consistence. The cerebrum was of the usual firmness, the cerebellum somewhat less so. The vertebral, basilar, and both carotid arteries were affected in their coats by atheromatous deposits. There was a considerable accumulation of fluid in the ventricles."

So far so good; we can associate the symptoms under which the patient died, either with the effusions of fluid both on the exterior and in the interior of the brain, or with the excited state of the cerebral circulation preceding such effusions; but what are we to say when told that the "acoustic and the vagus nerves were perhaps a *little softer* than natural; that the optic nerves were remarkably small, especially in relation to the large eyeball. The trochleares nerves were thinner than usual."

Will any experienced anatomist offer an opinion upon the relative hardness and softness of the seventh or eighth pair of nerves? or as to the normal size of the nerves of special sense? We do not hesitate to affirm that it is absolutely impossible, and that all such remarks, pretending to an accuracy which does not exist, are positively injurious to the advance of science. From a multitude of similar remarks we extract the following:—

"The pineal body was somewhat harder than in its natural state, the surrounding vascular structure (velum interpositum) more luxuriant and in a state of incipient hypertrophy, so that," says Dr. Bergmann, "according to conclusions formed from my repeated observations, deadening of the mental faculties would have ensued had life been much prolonged."

Are we to believe that this serious result (*Seelen-betaübung*) is an inevitable consequence of a condition of the pineal body and *velum interpositum*, absolutely not recognisable to the generality of those who have passed their lives in dissections and *post-mortem* examinations? A pineal body *harder than natural!* An *hypertrophied velum interpositum!* English anatomical acumen must be at a low ebb, for it would hesitate ere pronouncing an opinion upon such observations as these, and deducing thence such important conclusions. After a very elaborate account of the fibrous striæ and eminences in the interior of the brain, parts which, hitherto of little or no physiological interest, have received most barbaric names, we are told that "the fibrous case, which in its normal state covers the corpus striatum as a delicate veil, was, together with the tænia semicircularis, thickened and hardened.

"We found then," says the author, "that in this brain most of the fine structures approached perfection, some reached it. That the tænia semicircularis had lost, through induration, its beautiful free fibrous structure, and that the flabellum, the radiation of its fibres, had from the same cause disappeared.

"After the investigations which I have carried on in man as well as animals; after the pathological results which, in consequence of disease of the corpora-striata and of the anterior lobes of the brain, so often manifest themselves in the form of loss of memory; considering the direct connexion which the tænia semicircularis has with the organs both of smell and of sight, the conclusion may be readily drawn that in this situation memory has, in part at least, its seat."

Among the insane there is no more common symptom of mental derangement than loss of memory; past events are quite forgotten; persons and place are alike not recognised, and any gleam of intelligence bears the character of a confused and distorted dream. And yet morbid changes in the corpora striata or tæniæ semicirculares are most uncommon. We refer to a series of post mortem examinations conducted by Mr. Lawrence, at Bethlem Hospital, and published in the earlier numbers of the Journal in support of this statement.

We agree with Dr. Nasse in the able article forming one subject of the present review, that, "The present views and arguments in favour of the special relations of particular parts of the brain, are not yet established upon strong proof: and that the spirited and eagerly-contested question of the localization of the mental faculties is still in so unsatisfactory a state as to give weight to the opinion entertained by many, that our practical knowledge of the physiology of the brain has not been in any way materially advanced through its means."

The greater part of Dr. Nasse's essay is devoted to the consideration of the phrenological views of Bouillaud, who ascribed to the anterior lobes the seat of the principal legislator of speech (den Sitz des principe législateur de la parole), and who believes that in these organs are located, not only the recollection of words, but also the power to articulate them, or to regulate the muscular movements by which speech is formed.

The proofs brought forward in support of this statement may be divided into positive and negative. The first are instances in which disturbance of the power of speech co-existed with a morbid condition of the anterior cerebral lobes. The second comprise those cases in which, with unimpaired speech, there has been found a morbid condition of other parts than the anterior lobes. The cases brought forward both by Bouillaud and by Belhomme in support of this hypothesis, derived in part from personal experience, but chiefly from the observations of others, amount to about 200; but, as Dr. Nasse observes, they are inconclusive as to the important deductions formed by the French physiologist. There is scarcely a sudden and serious lesion of the brain in any part which is not followed by impairment of articulation; and the same phenomenon has been noticed when, with disease of other parts of the brain, the anterior lobes have been quite healthy.

To the striking examples brought forward by Andral and Cruveilhier, may be added the instances of loss of speech from disease of the middle lobe by Abercrombie; of the posterior lobe, by Rochoux; of the corpus striatum, by Serres; of the optic thalamus, by Bright; of the corpus callosum, by Abercrombie; of the cerebellum by Lallemand. Cruveilhier has recorded two cases, showing that the power of speech may be retained with extensive disease of the anterior lobes. In the first, the patient was a weak-minded unmarried female, aged fifty; the anterior lobes had entirely disappeared, and the space was occupied by a cyst. In the second, both anterior lobes were atrophied and softened by a large cancerous growth. Rochoux saw an instance in which the patient retained the power of utterance with effusion of blood into both anterior lobes; and Velpeau imparted to the Academy of Medicine in Paris, 1843, the case of a very talkative barber in whom there was discovered, after death, a scirrhous growth springing from the anterior part and inner surface of the skull, occupying the whole of the place of the right lobe, and a considerable portion of that of the left. In the Gazette Méd., of Paris, Dr. Nasse met with two cases of perforation and destruction of the anterior lobes by bullets, combined with unimpaired speech; and Jobert relates the case of a man who, with fracture of the frontal bone, and destruction of the anterior lobes, fell into a state of delirium, and spoke and sang most vehemently.

Dr. Nasse's Essay is full of information, and may be most advantageously perused by those who seek, in the present state of knowledge, to localise the cerebral functions. We now proceed to extract

### AN ACCOUNT OF THE ASYLUM FOR LUNATICS AT ERLANGEN, BY DR. SOLBRIG.

Professor Dr. Solbrig has given an interesting account of this asylum in the "Allgemeine Zeitschrift für Psychiatrie," vol. 8. This is a provincial institution, acknowledged to rank the first in Bavaria, and is established for the reception both of those cases which may be benefited by treatment, and of those which are incurable.

The directing physician is, in its full and extended sense, the director of the institution. There are no conflicting authorities fighting a petty battle for precedence; but there is unity of will—unity and precision in the carrying out of whatever is necessary for the good of the institution, and the welfare of its inmates.

As many of the asylums in England are officered under the mark, it may be as well to give the list of functionaries considered sufficient at Erlangen to preside over 150—160 patients. There is the director; a steward; one assistant physician; a head keeper in both male and female department; a book-keeper; an accountant; a cook and assistants; a washing establishment; a gardener; two porters; a messenger; housemaid; fourteen keepers for the insane men, and eleven for the insane women. Beside these, there are private attendants for special cases; a catholic and a protestant clergyman; a tutor and music-master; a catholic and a protestant organist; and also a clinique of psychiatry. There is free access to the establishment; nothing requires concealment, as the cases are regarded instances of disease which may befal any one; and which, with proper appliances and scientific treatment, admit mostly of alleviation, and sometimes even of cure. Have we in England advanced the knowledge of this interesting and important subject by shutting the doors of our asylums against the public gaze, and committing the inmates to a prison? We fear not. Until quite lately, when the dawn of enlightenment first broke through the mist which overshadowed the dwellings of the insane, to whom was this important trust committed? Not to physicians eminent for their high scientific acquirements and moral qualifications, but to persons who, without any claim to public confidence; without any wish or aspiration to advance their science, vegetated upon the proceeds of a huge lodging-house.

In the institution at Erlangen there is found a permanently greater proportion of males as compared with females in the ratio of two to one. There are always twice as many men as women; and the conclusion which Dr. Solbrig draws from this is, not that insanity is more common in the one class, but that females are more readily and easily taken care of than males in their own homes or in private asylums. He points out how difficult it is to arrive at satisfactory statistical conclusions from circumstances such as this.

In the department of incurables, the chief cases are those of fatuity (blödsinn), and partial crazedness (partielle verrücktheit), but there were seen very few cases of general paralysis. In the department for the treatment of the curables (heilanstalt) the predominant primary forms were mania (tobsucht) and melancholy. It was interesting to observe how politics took a place among the psychical causes of insanity, especially as illustrated by the events of the past year, 1850.

Many a victim was sent from this cause to the asylum at Erlangen, the manifestations of derangement assuming a very striking variety.

"Here heard we," says the author, "the speeches of the raging Jacobin; here saw we the fear of those threatened with loss of life and of property: we witnessed the arrogance of the violent democrat, who threatened every moment to take our institution by storm, and who lived to see more than once the declaration of a state of siege as the ordinance of a military commission. All in harmony with existing events. The last affected were mild doctrinists, who had dreamed themselves into the legislature, and who brought forward a whole code of laws, which were original enough, though perhaps rather too copious to admit of analysis here."

From the list of *post-mortem* examinations, amounting to forty-four inspections, we learn that about one-third died of tuberculosis of the lungs.

In speaking of treatment, Dr. Solbrig mentions the high importance of placing the patient under circumstances where every source of excitement and irritation is removed. It cannot be too often repeated how rarely in mania there exists a state of congestion of the vessels of the brain, requiring active depletion; or rather, it might be said, in how great a proportion of cases is it found that there is manifested a state of irritation of the cerebro-spinal centre, for the cure of which loss of blood is a fruitless measure, and under certain circumstances of great nervous exhaustion apt to be followed by even a fatal result.

Against the proper and healthy exercise of the psychical functions, there is no greater enemy than hunger, and an empty lymphatic system. Excess of nutrition is not a common cause of insanity; hence depletions of any kind must be demanded only in exceptional cases.

Experiments with the administration of opium seem to have been carried out to a considerable extent, but not with so happy a result as in some cases by Engelken. In doses of a quarter to half a grain, it did not succeed in changing the disposition, and improving the spirits of those affected with melancholy.

As regards larger doses, Dr. Solbrig had no fear, on cases of excitement affecting non-plethoric persons, of giving four, six, or eight grains of pure opium at a time, twice or thrice in twenty-four hours, and to continue the practice for three consecutive days.

"We may thus," he says, "procure for the patient sleep, and momentary repose, which is always worth something; but in one case only did I witness a striking beneficial result. It was that of a maniacal countryman, who for fourteen days before his admission had been suffering from the most furious excitement and impulse to destroy. A dose of six grains of opium sent him into a profound sleep, attended by profuse perspiration. Upon waking he was perfectly quiet, and soon passed into convalescence."

No good results ensue from prolonging these large doses over a space beyond three days; perhaps such practice might render probable the chance of collapse. Dr. Solbrig prefers as an opiate the ext. cannab. indic. (hachisch), especially in periodical mania, where it is complicated with convulsive and chorea-like movements, or with inveterate epilepsy. It is given in doses of one to four grains of the extract two or three times in the twenty-four hours.

In powerful subjects the prolonged use of the warm bath from three to six hours is recommended. This may be combined with the simultaneous flow of cold water over the head by directing a fine stream to pass upon a broad linen compress.

In females where symptoms of nymphomania are present, the daily use of the hip-bath for two hours is stated to be useful.

No satisfactory result has followed the administration of sulphuric-ether, or of chloroform. The employment of ether seems to be contra-indicated, inasmuch as it favours beyond all doubt an hyperæmic condition of the brain, far more than is desirable. In mania, it has no effect. After the immediate influence of the inhalation has passed away, perhaps in two or three minutes, the symptoms recur with their former severity.

Upon the use of chloroform, Dr. Solbrig speaks as one whose opinions as to its efficacy in the treatment of some cases, are not ultimately formed. He uses it at present chiefly to calm violent paroxysms, or to enable patients to undergo surgical operations without pain.

"The greatest triumph was in a case where a patient obstinately refused to have his *hair cut*. There it afforded radical assistance. It is certainly the mildest means of coercion."

Dr. Solbrig does not approve of the total absence of bodily restraint, as carried to its fullest extent in the English institutions. He thinks that there is but little to boast of, if the practice is followed by injuries inflicted by excited patients on themselves, their fellow sufferers, or on their keepers. Perhaps we are not quite wise in abandoning in every case such restraint as may prevent attempts at self-destruction, for it is impossible, unless the number of attendants be very large, that a sufficiently strict guard should be kept upon all patients during every hour of the day. Several cases have come before our notice, in which very serious accidents have occurred in institutions, where the non-restraint system existed in full force, and among other evils is that of the keepers feeling it sometimes necessary to employ their bodily strength rather freely in self-defence. We have met with fractures of the skull, of fractures of the sternum, and of other bones, in patients reported to have died suddenly. Such injuries have doubtless been inflicted during some severe struggle, when the patient, determined upon a mad attempt, has threatened violence to all who would venture to thwart him. We have reason, however, to be thankful to those who have pioneered the way to the non-restraint system; it is gradually becoming more and more extended; the comfort and welfare of the sufferers receive daily more and more attention; the calamity is no longer regarded as a family stain, demanding the immolation of one of its members.

May we not hope that ere long society will adopt general means of yet further restricting the evil. The disease attacks the weak rather than the strong; the feeble constitution threatened with tuberculosis rather than the robust and plethoric. Intermarriages within too close a degree are sure to produce a large proportion of inferiorly developed minds. Such persons may, it is true, pass through life in the respectable performance of its ordinary duties; but should at any period excitement ensue, caused by politics, religious enthusiasm, or excess of business, the brain is found incapable of reacting against the stimulus; and it loses its balance, which is never subsequently regained.

An article on "Court Fools" is worthy of analysis. The history of court fools cannot be passed over in a complete history of Psychiatry, because among them there were found at all times not only professional fools and buffoons, but also real madmen and imbeciles: to conduct the inquiry here is interesting as regards the manners of the age; but it is also of importance for practical medicine; for it is worth knowing how, in different periods, poor mentally deranged beings were treated. It is also remarkable, that in Eastern countries, their number always was, and is still, less than in Western countries, and that in the former the insane are honoured and well cared for as specially favoured by God, while in the latter, even in our days, they have been barbarously treated; mental derangement and sin being considered alike. Here history gives the best and most beautiful practical lesson.

The "History of Court Fools, by H. F. Flögel, Professor in the Ritter Academie, at Liegnitz," is the subject of the following essay.

The history of fools and of buffoons can be traced back to the dark days of antiquity; princes and commoners, high and low, delighted in them; the part was played by pedantic men of talent, rather than by real madmen, and there were towns and whole districts which had the unenviable repute of being the natural soil of fools. Thus the town of Troyes enjoyed the peculiar privilege of providing court fools for the kings of France. In Troyes, is still preserved a letter from Charles V. of France, in which he announces the death of his fool, and requires the Burgomaster of Troyes to send him another, according to ancient custom. The German Emperor, Charles V., son of the imbecile Jane of Spain, said of the Germans, that they did not seem to be clever, and really were not so.

The Emperor Rudolf II. could not bear a jester in his presence; probably because he was much inclined to melancholy.

Richardson brings the origin of fools from Eastern countries, on account of the particular esteem in which the deranged were there held. The sayings of fools were reverenced as inspirations, and it was from this cause that they were allowed the freedom of carrying their satires so far. Owing to this circumstance, cunning and designing persons took the opportunity of "playing the fool,"—they could speak out the truth without fear of punishment.

Those who translate the word "morio" by court fool, make a great mistake, for morio meant by the Romans, a man who was deformed. The morio had a humped back, bandied legs, a great shapeless head, a long pendulous nose, or remarkable features; in a word, the morio (morris dancer) was a mis-shaped being; and probably besides, either weak minded or an absolute fool. The morio was so constituted, both bodily and mentally, as to excite laughter, and was on that account taken advantage of and misused as a fool. The tasteless pleasure taken by the degenerated Romans in such unfortunates went so far, that there was held in Rome an express market for fools—Forum Morionum—at which the dealers publicly exposed for sale the morios, whom they had gathered from all parts of the world, and obtained for them large prices. A thoroughly deformed morio often fetched a thousand florins. Martial viii. 13; "Morio dictus erat; viginti millibus emi." Further, "Plutarchus de Curiositate." Pliny and Martial both say that the morions were stupid and imbecile. Plinius, lib. ix. ep. 17; Martial, xiv. 210; Martial, viii. 13; xii. 95.

Court fools are mentioned in the East as early as the eighth century. Such a being is found to have existed at the court of King Attila. At the court of the Khalifh Harun-al-Raschid there lived a man whom the Mahomedans used to regard as one inspired or insane. The Sultans of Turkey have, or used to have, such persons about their court, whereof there can be seen a full account in a work by Nicholas Höniger—"Die Hofhaltung der türkishen Kaiser," Bazel, 1596, tom i., s. 39.

The jester (Lustigmacher) at the courts of the German Princes, does not seem at all times to have been able to bear with sufficient patience the tricks which were so often played upon him; probably because the difference between a madman and a mountebank was not sufficiently well understood. As the Duke Lewis of Bavaria, September 16, 1231, was taking a walk after dinner to Kehlheim, over the Danube bridge, he provoked his court fool, Stich or Stichius, to such a degree, that the man became mad and murdered the Duke with a bread-knife. The murderer was destroyed on the spot by the attendants. (Aventinus Annal. Bojor. lib. vii., c. 3., p. 634, Ex. Editione Gundlingii). Some relate this murder in a different way; but Aventinus's view seems the simplest and the most probable. The Baron (Freiherr) Jacob Paul Von Gundling acted the part of fool at the court of King Frederick William I., the name Gundling being given to him from the unlimited inclination forward of the trunk, which gave a creeping aspect to his gait. Thus came his stiff comic look, which he himself produced, to render himself an object of mirth. He could never be satisfied in drinking wine, and his constant complaint was thirst. That Gundling was a clever scholar and contributed something to history is well known. A psychological delineation of this extraordinary man would be of interest both in theory and practice. Gundling died 1731; at the examination after death there was fonnd a perforating ulcer of the stomach.

A certain Kornemann from Halberstadt appeared about the time of Gundling; he called himself Cron-Kornemann, was completely deranged, but nevertheless enjoyed himself. Subsequently being in want of money, he cut his throat, but the wound was not fatal; he recovered, and was put into confinement.

The last fool that we shall mention here was one at Schweidnitz. Boteslaus II., the Little, Duke of Schweidnitz, died 1368, and with him ended that line of the house, his son having been murdered by a fool. In an old manuscript we read that "Duke Boteslaus and his wife Agnes had a son, whom the fool, before the father's death, killed with a tile at the castle of Bolkenhayn, in consequence of the Prince having vexed him and excited his anger. The fool was beheaded."

We see that in the thirteenth and fourteenth centuries the doctrine of irresponsibility of action was not in vogue; this was certainly an omission in the law. In the present day we are inclined to pronounce the malefactor "irresponsible" upon very insufficient grounds; not, forsooth, as a manifestation of Christian love, but that advocates may show their sharpness and learning, and that writers may glorify themselves in the journals. The false humanity of the present age may bring Corsican vengeance into the land. As regards France, where matters are usually driven into extravagance, it is remarked that court fools were formally attached to the court as "fous en titre d'office."

In the American Journal of Insanity, there is related a case of "pyromania," which bears somewhat upon the preceding remarks. A youth, of fifteen, who had abstracted money from several letters, set fire to some sheds, having been instigated to the act by another. The reporter of this case makes the concluding remark, that most "pyromaniacs" observed by him had manifested other insane and dangerous tendencies; they had stolen or murdered; and he concludes that the passion to set fire to objects cannot be regarded as so distinct a form of insanity as to require a special name. If such a young rogue were to escape punishment on the grounds of insanity, and the decision were to be taken as a precedent, it would be wise for us in all consistency to shut up the courts of justice, and to employ the judge's salaries in enlarging lunatic asylums over the whole land.

